# Alternative Proteins on the Polish Plate: Consumer Acceptance, Current Challenges, and Future Perspectives

**DOI:** 10.3390/foods15173061

**Published:** 2026-08-29

**Authors:** Anna Choręziak, Dominika Sikora, Piotr Rzymski

**Affiliations:** 1 Department of Environmental Medicine, Poznan University of Medical Sciences, 60-806 Poznan, Poland; anna.choreziak@ump.edu.pl (A.C.); dsikora@ump.edu.pl (D.S.); 2 Doctoral School, Poznan University of Medical Sciences, 60-806 Poznan, Poland

**Keywords:** novel foods, plant-based, insects, cultured meat, microalgae, mycoproteins, central Europe

## Abstract

The transition toward more sustainable diets has increased interest in alternative protein sources, including plant-based meat substitutes, cultured meat, edible insects, microalgae, and microbial fermentation products. Their successful adoption depends not only on technological advances and market availability but also on consumer acceptance shaped by cultural, sensory, psychological, ethical, and socio-demographic factors. This narrative review synthesizes evidence on consumer acceptance of alternative proteins in Poland, a country characterized by strong culinary traditions, high attachment to meat, and selective openness to food innovation. The evidence base is fragmented and uneven across product categories. Plant-based alternatives are the most established but face barriers related to price, sensory expectations, perceived nutritional quality, and concerns about ultra-processing. Cultured meat shows moderate acceptance, limited by perceived unnaturalness, safety concerns, and low familiarity. Edible insects remain the least accepted due to disgust, food neophobia, and cultural barriers. Evidence on microalgae and mycoproteins is scarce, while consumer responses to fermentation-derived proteins are largely unexplored. Overall, acceptance varies by product type and requires targeted improvements, culturally sensitive communication, and more robust research.

## 1. Introduction

The global food system is under increasing pressure from population growth, climate change, environmental degradation, and the need to ensure long-term food security [1]. Protein is an essential macronutrient, providing amino acids necessary for growth, maintenance, and repair of tissues, as well as the proper function of many physiological processes. Its primary dietary sources include animal-derived products, such as meat, fish, eggs, and dairy products, as well as plant products, including legumes, grains, nuts, and seeds. However, protein sources vary in their nutritional value. Animal proteins are characterized by a favorable essential amino acid profile, whereas plant sources may require combining different products to achieve a well-balanced amino acid profile [2]. Significant differences also apply to the environmental impact of individual protein sources. Conventional livestock production contributes substantially to greenhouse gas emissions, land and freshwater use, biodiversity loss, and antimicrobial resistance, prompting growing interest in more sustainable sources of dietary protein [3,4]. As a result, a wide range of alternative proteins (i.e., plant-based meat substitutes, cultured meat, edible insects, microalgae, microbial fermentation products, and single-cell proteins) have emerged as promising components of future food systems and a potential part of postulated food system transformation [5,6,7,8,9]. Beyond their environmental benefits, these innovations may also improve resource efficiency and address ethical concerns related to animal welfare [9,10].

Importantly, the transition toward more sustainable protein consumption depends not only on technological development and market availability but also on public acceptance. The acceptance of alternative proteins is a complex outcome of interacting psychological, social, cultural, ethical, religious, and culinary factors. It is therefore not only shaped by general attitudes toward food but also by perceptions of specific products. Different alternative protein sources evoke distinct responses; for example, cultured meat, edible insects, plant-based meat analogs, and microalgae-based products may differ substantially in perceived naturalness, safety, taste expectations, and familiarity [10,11]. As a result, acceptance varies not only between countries but also between individual protein alternatives. These differences suggest that communication strategies aimed at promoting sustainable dietary transitions cannot be universally applied across contexts. Instead, they should be tailored both to the cultural setting and to the specific characteristics of the alternative protein being promoted [12,13,14].

Consumer responses to food innovation are deeply embedded within cultural and social contexts. Some societies demonstrate relatively high openness to novel foods and food technologies, while others exhibit stronger attachment to traditional dietary practices and greater reluctance toward unfamiliar products. A higher willingness to adopt novel foods might be facilitated by greater technological progressiveness, as in Singapore, or by greater food security caution, as in India, in contrast to Western meat-centered societies, which take food security for granted [14]. Consequently, acceptance of innovative protein sources varies considerably across countries and populations. Understanding these cultural differences is essential for predicting consumer behavior and designing effective strategies to facilitate sustainable dietary transitions [15].

In countries such as Poland, the adoption of alternative proteins must be considered against the background of strong attachment to conventional meat and traditional dietary practices. Meat occupies a central position in Polish cuisine, and per-capita consumption remains among the highest in Europe [12,16]. This strong culinary tradition coexists with growing awareness of environmental sustainability, animal welfare, and health considerations, contributing to increasing interest in vegetarian, vegan, and especially flexitarian dietary patterns, particularly among younger generations [17]. These trends create both opportunities and challenges for the introduction of alternative protein sources, as their acceptance may be simultaneously facilitated by shifting dietary norms and constrained by attachment to conventional meat consumption. High meat consumption is also characteristic of other European countries, where intake exceeds dietary recommendations [18,19]. Therefore, examining consumer attitudes towards alternative protein sources in Poland may provide insights relevant to other European populations facing similar challenges.

Available evidence nevertheless suggests that Polish consumers do not respond uniformly to food innovation. Greater food neophobia and reluctance toward technologically modified or unfamiliar products have been reported among older individuals and those with lower educational attainment and income, whereas younger and more highly educated consumers generally demonstrate greater openness to novel foods [20]. Acceptance also appears to depend strongly on the type of alternative protein. Plant-based products may benefit from relative familiarity, whereas cultured meat, edible insects, and fermentation-derived products may elicit concerns related to unnaturalness, safety, disgust, technological intervention, or incompatibility with established culinary practices [21].

Against this background, the present narrative review aims to synthesize and critically evaluate evidence published over the last decade on consumer acceptance of alternative protein sources in Poland. The review first briefly introduces the main categories of alternative proteins, including plant-based meat substitutes, cultured meat, edible insects, microalgae, mycoproteins, single-cell proteins, fermentation-derived products, and other emerging protein sources. It then examines Polish studies addressing consumer attitudes, willingness to try or purchase, perceived benefits and risks, and the determinants of acceptance across these categories. Particular attention is given to sociodemographic, psychological, cultural, sensory, and behavioral factors. By integrating evidence within a single national context, the review seeks to identify common and category-specific patterns of acceptance, methodological limitations, research gaps, and implications for future research, consumer communication, and culturally appropriate strategies supporting sustainable dietary transitions.

## 2. An Overview of Alternative Protein Sources

### 2.1. Cultured Meat

Cultured meat (also referred to as cultivated, cell-based, or lab-grown meat) is produced by culturing animal cells in vitro under controlled conditions rather than by raising and slaughtering livestock [18,19]. The production process involves isolating stem or satellite cells from animals, proliferating them in nutrient-rich culture media, and differentiating them into muscle and other tissues in bioreactors, often employing tissue engineering techniques and 3D printing [20,21]. The resulting product is intended to replicate the sensory, nutritional, and functional characteristics of conventional meat while substantially reducing the need for animal farming. The protein content and amino acid composition depend on cell type, culture conditions, and composition of the final product. However, emerging data indicate that cultured meat products contain lower amounts of protein and essential amino acids than their conventional meat counterparts [22,23]. Commercial products include cultivated chicken (Singapore and the USA), cultivated salmon (the USA), and cultivated Japanese quail (Australia and Singapore). Consumer acceptance of cultured meat in Europe appears to be moderate. Concerns regarding unnaturalness and technological risks remain barriers to wider acceptance [24,25,26].

Cultured meat has attracted considerable attention because of its potential to reduce greenhouse gas emissions, land use, water consumption, and animal suffering associated with conventional livestock production. Yet, currently, energy and environmental impacts considerably vary depending on cultured meat type, e.g., production of cultured chicken meat requires more energy than conventional chicken meat, and thus by extension generates higher GHG emissions [27]. Consequently, the outcome of the environmental burden of cultured meat will depend on the progress in optimizing the production process of cultured meat as well as the pace of shifts toward decarbonized energy sources [28]. From another perspective, cultured meat may also improve food safety by reducing exposure to zoonotic pathogens and limiting the use of antibiotics in animal production [22,23,24,25]. However, commercialization depends not only on overcoming technological and economic barriers but also on obtaining regulatory approval [26]. The first authorization for a cultured meat product was granted by the Singapore Food Agency in 2020 for cultivated chicken, followed by approvals from U.S. regulatory authorities for cultured chicken in 2023 and pre-market authorization of cultured salmon by the U.S. Food and Drug Administration in 2025 [27,28,29]. In Europe, applications for cultivated foie gras and cultured animal fat were submitted under the Novel Food Regulation in 2024 and 2025, reflecting increasing regulatory engagement with these products [30,31]. Despite these advances, cultured meat remains at an early stage of market development. Recent re-evaluations of regulatory submissions indicate that, although currently approved products generally meet established safety requirements, variability in nutritional composition across production batches underscores the need for further optimization and standardized manufacturing processes [32]. Moreover, commercialization efforts and research remain concentrated primarily in Europe and North America, where consumer interest is frequently driven by concerns regarding environmental sustainability, animal welfare, and reducing reliance on conventional livestock production [29].

### 2.2. Plant-Based Meat Substitutes

Plant-based meat substitutes comprise a diverse group of products formulated from plant-derived protein sources, including soy, pea, wheat, faba bean, oat, rice, and other legumes and cereals [30,31]. Through combinations of protein isolation, extrusion, flavor development, and texturization technologies, these products are designed to mimic the appearance, texture, flavor, and cooking properties of conventional meat [32]. Continuous technological advances have considerably improved their sensory characteristics and expanded their availability in retail markets [33,34]. Their protein content and amino acid composition vary considerably depending on the formulation. Although plant proteins provide all essential amino acids, individual sources may be limited in specific amino acids, particularly methionine in legumes and lysine in cereals, and their digestibility in general is lower than that of animal proteins; however, protein blending and processing technology improve their nutritional profile [35,36]. Plant-based proteins are widely commercialized in the form of burgers, sausages, nuggets, mince, and other analogs and represent one of the most commercially developed categories of alternative proteins. In 2020, it was estimated at around 2% of the animal protein market. The percentage of protein derived from algae, insects, microbiologically fermented products, and cultured meat is unknown, but is estimated to be a fraction of this total, both globally and at the EU level [27]. A survey conducted in European countries shows that 28% of respondents reported consuming plant-based food alternatives at least once a week, while 43% intended to increase their consumption and purchase [37].

Owing to the multiple processing steps involved in protein extraction, fractionation, reformulation, and industrial manufacturing, most commercially available plant-based meat substitutes are classified as ultra-processed foods according to the NOVA classification system [37]. This classification has generated considerable scientific and public debate. Critics argue that extensive industrial processing, long ingredient lists, and the use of additives such as stabilizers, emulsifiers, colorants, and flavorings may negatively influence consumer perceptions and are sometimes shown to be associated with adverse health outcomes in observational studies [38,39]. Conversely, many researchers emphasize that the degree of processing should not be considered in isolation, as nutritional quality depends primarily on the overall composition of the product, including protein quality, dietary fiber, micronutrient fortification, sodium content, and the types and amounts of fats used [37,40]. From this perspective, well-formulated plant-based meat substitutes may offer nutritional advantages compared with certain processed meat products despite their classification as ultra-processed foods.

Among alternative proteins, plant-based meat substitutes currently represent the most mature and commercially successful category in many countries [9]. Their widespread availability, familiarity, and compatibility with flexitarian dietary patterns contribute to relatively high consumer acceptance. At the same time, consumers differ considerably in their perceptions of nutritional quality and degree of processing. While many view plant-based products as healthier and more environmentally sustainable than meat, others express concerns regarding long ingredient lists, the use of additives, and the extent of industrial processing [41]. Consequently, purchase decisions are influenced not only by sustainability considerations but also by taste, price, convenience, nutritional expectations, and trust in manufacturers [42]. Recent studies indicate that perceptions of ultra-processing may negatively influence consumers’ willingness to purchase plant-based meat substitutes, even among individuals who acknowledge their environmental benefits, highlighting a persistent tension between sustainability-oriented motivations and preferences for minimally processed foods [39,43].

### 2.3. Edible Insects

Edible insects are among the oldest and most widely consumed alternative protein sources globally, with more than 2000 species traditionally consumed across Asia, Africa, Latin America, and Oceania [38]. Species commonly considered for human consumption include crickets, mealworms, grasshoppers, locusts, and black soldier fly larvae. In the European Union, the commercialization of insects for food purposes is regulated by novel food legislation, and up to mid-2026, four species were authorized for consumption: house cricket (*Acheta domesticus* L.), larval forms of yellow mealworm (*Tenebrio molitor* L.), migratory locust (*Locusta migratoria* L.), and larvae of buffalo worm/lesser mealworm (*Alphitobius diaperinus* Panzer) [39]. In the EU, edible insects have reached the market whole, frozen and dried, or as powders and ingredients incorporated into other foods like chips and other snacks [40]. Despite their relatively advanced technological development, their contribution to the European market remains small. Consumer acceptance is one of the major barriers to their wider commercialization, particularly due to food neophobia and disgust. Acceptance is generally higher when insects are incorporated into familiar foods and are not visually recognizable [41].

Insects are characterized by high protein content (from 20–76% of dry matter), favorable amino acid profiles, and substantial amounts of unsaturated fatty acids, vitamins, and minerals, making them nutritionally comparable to many conventional animal-derived foods [42,43,44]. The total protein digestibility varies between species, and for migratory locusts and yellow mealworms ranges from 75 to 85% [45]. From an environmental perspective, insect farming generally requires significantly less land, water, and feed than conventional livestock while producing lower greenhouse gas emissions [46,47,48]. Insects also exhibit high feed conversion efficiency and can be reared using certain organic side streams, contributing to circular bioeconomy concepts [49]. Despite these advantages, acceptance in Western countries remains relatively low [50,51]. Food neophobia, disgust, unfamiliarity, perceived safety concerns, and cultural norms consistently represent the principal barriers to consumption, whereas processing insects into less recognizable forms, such as protein powders or ingredients incorporated into familiar foods, tends to improve consumer acceptance [51,52,53].

### 2.4. Microalgae

Microalgae are photosynthetic microorganisms capable of producing substantial amounts of protein, lipids, carbohydrates, vitamins, pigments, and bioactive compounds [54]. Species such as cyanobacteria *Arthrospira* spp. (commercially known as Spirulina) and green algae *Chlorella* spp. have long been commercially cultivated for food and dietary supplements and are among the best-known microalgae intended for human consumption [55,56]. Depending on the species, cultivation conditions, and harvesting stage, microalgal biomass may contain approximately 40–70% protein on a dry-weight basis while providing a balanced amino acid profile, polyunsaturated fatty acids, dietary fiber, minerals, antioxidant compounds, and selected vitamins [57,58]. Protein digestibility may be reduced by the presence of cell walls, while processing can substantially improve protein accessibility. In addition, clinical trials have suggested their immunomodulatory, antihypertensive, antilipidemic, and hypoglycemic effects [59,60,61]. Microalgae are commercialized as dried biomass and powder used in dietary supplements and functional foods, and incorporated into products such as pasta and beverages. Microalgae-based foods are not yet widely familiar to European consumers, and their willingness to try them has been reported as moderate [62]. Nevertheless, they generally appear to accept microalgae as food ingredients, particularly when they are incorporated into familiar food products at relatively low inclusion levels [63].

Microalgae have attracted considerable interest as a sustainable protein source because they can be cultivated on non-arable land with substantially lower freshwater requirements than conventional agriculture while exhibiting high biomass productivity per unit area [64]. Their cultivation can also contribute to carbon dioxide capture and may be integrated into circular bioeconomy systems by utilizing industrial emissions or nutrient-rich waste streams [65]. Furthermore, cultivation in controlled photobioreactors enables year-round production independent of seasonal fluctuations, though it requires substantially increased energy supply [66].

Despite these advantages, several technological, economic, and safety-related challenges continue to limit the wider use of microalgae in human nutrition. Production costs remain relatively high, particularly for closed cultivation systems that ensure consistent quality and minimize contamination [67,68]. Sensory characteristics, including the intense green color, earthy or marine flavor, and distinctive aroma, may reduce consumer acceptance and often require formulation strategies to improve palatability. In addition, the digestibility and bioavailability of nutrients may vary among species, as robust cell walls can limit nutrient release unless appropriate processing methods are used [69,70]. Food safety considerations are equally important. The chemical composition of microalgae is highly dependent on cultivation conditions, and poorly controlled production systems may lead to contamination with toxic metals, pesticide residues, pathogenic microorganisms, or cyanotoxins, and lead to health issues [71,72,73]. The use of wastewater or industrial side streams as nutrient sources, although environmentally attractive, requires strict monitoring to prevent the accumulation of undesirable contaminants in the harvested biomass [74]. Consequently, standardized cultivation protocols, comprehensive safety assessment, and regulatory oversight are essential to ensure the quality and safety of microalgal products intended for human consumption. Although *Spirulina* and *Chlorella* have established safety records and are widely commercialized, many other microalgal species remain subject to regulatory evaluation before broader incorporation into the food supply [75].

### 2.5. Microbial Fermentation Products

Microbial fermentation comprises traditional, biomass, and precision fermentation. In the case of meat alternatives, the former comprises traditionally fermented foodstuffs, such as tofu or tempeh, which are classified as plant-based meat alternatives [76,77], while biomass fermentation and precision fermentation tend to be categorized as more novel meat alternatives owing to their commercialization maturity [27].

Biomass fermentation refers to single-cell proteins (SCPs), which are protein-rich biomass produced from microorganisms such as bacteria, yeasts, fungi, and microalgae cultivated under controlled conditions [78,79,80]. Advances in industrial biotechnology have enabled microorganisms to utilize a wide range of substrates, including agricultural by-products and industrial side streams, providing opportunities for highly resource-efficient protein production [78,80,81]. Depending on the production system, SCPs may be incorporated directly into foods or used as ingredients in processed food products [78,82,83]. Among SCPs, the best-known commercial example is derived from *Fusarium venenatum*, which forms a fibrous biomass resembling the texture of muscle tissue after processing [27,84]. Mycoprotein products typically contain high-quality protein, dietary fiber, relatively low levels of saturated fat, and several essential vitamins and minerals, making them nutritionally attractive alternatives to meat [27]. One of the best-characterized microbial proteins is the Quorn™ mycoprotein product, which contains 45–54% proteins, and has a digestibility of 65–84%. It is already commercially available in Europe as burgers, nuggets, mince, and slices. Precision fermentation is increasingly being developed for the production of dairy and egg proteins. Nevertheless, their overall contribution to the European market remains small. Consumer acceptance appears moderate to low and depends on familiarity with the product [85,86].

The production of mycoproteins generally requires substantially fewer natural resources than conventional livestock farming while generating comparatively low greenhouse gas emissions. Because mycoprotein products often resemble meat in texture and culinary applications, they have achieved relatively high acceptance among consumers seeking meat alternatives [27]. Nevertheless, broader adoption may still be influenced by perceptions regarding processing, product familiarity, pricing, and individual preferences for minimally processed foods. Rare allergic reactions have also been reported, highlighting the importance of appropriate labeling and post-market surveillance.

Closely related are fermentation-derived proteins produced using precision fermentation technologies, in which genetically engineered microorganisms synthesize specific proteins, such as dairy or egg proteins, without requiring animal agriculture [27,87]. These technologies have emerged as promising approaches for producing functional food ingredients with potentially reduced environmental footprints, although the magnitude of these benefits depends on factors such as energy source, carbon substrate, purification requirements, and production scale [87,88]. Despite considerable technological potential, consumer acceptance remains uncertain and appears strongly influenced by perceptions of genetic engineering, naturalness, food safety, sensory quality, and trust in biotechnology and food manufacturers [89,90,91]. As these products move toward broader commercialization, transparent communication regarding production methods, regulatory oversight, allergenicity, and potential benefits will likely play an important role in shaping public acceptance [83,90,92].

## 3. Methodology of Review

To identify relevant literature for this narrative review, a structured literature search was conducted in Google Scholar, Scopus, Web of Science, PubMed, and Polish database BazEkon, Repozytorium Otwartych Publikacji Naukowych OPEN. The search covered scientific publications published in Polish or English between 2015 and 2025. The review focused on studies addressing consumer acceptance, perceptions, attitudes, willingness to consume or purchase, and related behavioral determinants concerning alternative protein sources among Polish consumers. The literature search was conducted between 30 January and 30 March 2026. A combination of keywords and Boolean operators was used to refine the search strategy. The main search terms included “Poland” OR “Polish” combined with “acceptance” AND “consumers”, together with terms referring to specific categories of alternative proteins. The following keyword groups were applied: cultured meat: “in vitro meat”, “cultured meat”, “cultivated meat”, “slaughter-free meat”; edible insects: “edible insects”, “insect-based products”; microalgae: “algae”, “microalgae”, “microalgal”, “Spirulina”, “Chlorella”; mycoproteins: “mycoproteins”, “microbial proteins”; plant-based alternatives: “plant-based”, “plant burgers”, “plant meat”, “plant alternatives”; and precision fermentation: “precision fermentation”, “microbial foods”, “microbial fermentation”, “microbial proteins”. Boolean operators such as AND and OR were used to combine broader population-related terms with product-specific terminology.

The screening process was conducted in two stages. First, titles and abstracts were screened to assess each publication’s relevance to the review topic. Publications were considered eligible if they reported original data, reviewed the literature, or provided relevant contextual information on Polish consumers and alternative protein sources. In the second stage, the full texts of potentially relevant publications were assessed in greater detail. The reference lists of included publications were also manually screened to identify additional studies that may not have been retrieved during the initial database search. Studies were included if they addressed consumer perceptions, attitudes, acceptance, willingness to consume or purchase, perceived barriers, or socio-demographic and psychological determinants of acceptance of alternative protein products in Poland. Publications were excluded if they did not concern Polish consumers; did not address alternative protein sources; focused exclusively on technological, nutritional, environmental, or regulatory aspects without any consumer-related component; or were outside the predefined publication period or language criteria. Duplicate records and publications with only marginal relevance to the aims of the review were also excluded. The initial screening of titles and abstracts was performed by one author and subsequently double-checked by another author. Full-text eligibility assessment was conducted using the same approach. Any uncertainties regarding inclusion were discussed between the authors until agreement was reached. In total, 34 publications meeting the initial search criteria were identified and considered during the screening process. Following full-text assessment, publications with limited relevance to the review objectives were excluded, while the remaining studies were included in the narrative synthesis.

Data extracted from the included publications included the type of alternative protein investigated, study design, study population, sample characteristics where available, key measures of consumer acceptance or perception, and the main findings relevant to Polish consumers. Given the heterogeneity of study designs, product categories, outcome measures, and populations, no quantitative meta-analysis was conducted. Instead, the findings were synthesized narratively according to the main categories of alternative proteins considered in the review.

## 4. Acceptance of Alternative Protein Sources in Poland

### 4.1. Overview of Studies

The available Polish literature on consumer acceptance of alternative protein sources is unevenly distributed across product categories. Our review identified 6 articles on cultured meat, 6 on plant-based meat alternatives, 19 on edible insects, 5 on microalgae, and 1 on mycoprotein. All of these studies are summarized in Table 1. Only three studies were published in 2017, but most were published between 2020 and 2026 and relied on survey-based quantitative designs, typically using self-administered questionnaires. Only one study included qualitative methods, and no mixed-methods studies were identified in the current evidence base. Most studies used non-representative samples and therefore did not adequately reflect the demographic structure of the Polish population. Although sociodemographic variables were frequently collected, their selection, categorization, and reporting differed substantially across studies, limiting direct comparisons between product categories. This methodological heterogeneity should be made explicit because it constrains the strength of conclusions that can be drawn about the Polish population as a whole.

Table 2 summarizes the main factors promoting consumer acceptance and the key barriers associated with each specific alternative protein source. However, some of the reviewed studies were conducted with relatively small samples, which may limit the representativeness and generalizability of their findings. Therefore, the identified drivers and barriers should be interpreted with caution.

### 4.2. Acceptance of Cultured Meat

Polish acceptance of cultured meat was assessed in five online studies using different self-designed questionnaires [21,25,95,100]. One of these studies was restricted to meat consumers [21]. Across the studies, women and participants with higher education were overrepresented [21,25,95], which is typical of online survey-based research [123], but limits the generalizability of the findings to the broader Polish population. Acceptance was most often operationalized as willingness to buy, try, or consume cultured meat and generally ranged from low to moderate [21,25,95]. One study assessed cultured meat acceptance by comparing it to conventional meat [100]. Familiarity with the concept of cultured meat was also low to moderate across studies [21,25,95], although strong skeptics constituted a minority in studies that assessed this subgroup [21,25]. The main incentives for accepting cultured meat among Polish consumers were perceived sustainability benefits and animal welfare [25,95], as well as expected good taste and an attractive price [95]. Conversely, the dominant barriers were safety concerns, perceived unnaturalness, and limited knowledge about cultured meat [21,25,95,100]. Poor taste, high price, and limited availability were also reported [95]. The available studies suggest that potential early adopters in Poland are more likely to be younger consumers [21,25], individuals already familiar with cultured meat [21,25,95], and those with greater knowledge of and interest in animal welfare and environmental issues [21,25].

Cultured meat is intended to reproduce the sensory and culinary attributes of conventional meat while reducing selected ethical and sustainability concerns associated with livestock production [124,125]. In principle, this positioning may reduce the perceived trade-off between hedonic gratification and value-driven motives [21]. This is particularly relevant for consumers who are willing to reduce meat consumption but are not ready to eliminate meat-like products from their diet [95]. Higher acceptance among Polish meat-eaters than among meat excluders may reflect the fact that many meat excluders have already adapted to meat-free diets and therefore have less need for products that mimic meat’s sensory and culinary roles [25].

At the same time, cultured meat depends on an unfamiliar production process, and this technological character may itself become a barrier to acceptance of novel foods, especially in contexts characterized by lower openness to innovation [126]. The production of cultured meat involves complex procedures and diverse protocols that are difficult for average consumers to understand [124,127]. This may partly explain why familiarity with cultured meat was more common among higher-educated Poles [95]. Production-related uncertainty can therefore reinforce barriers to acceptance, particularly because consumers often respond more strongly to negative than to positive information [127,128]. Consistently, greater familiarity with cultured meat was associated with higher acceptance in Poland [21,25], whereas the most frequently reported barriers were perceived unnaturalness, safety concerns, limited knowledge, and disgust [21,25,100].

Perceived naturalness is a broad and partly abstract food attribute. It may refer to product composition, such as the absence of artificial ingredients, preservatives, additives, chemicals, or genetically modified organisms; to production characteristics, such as minimal processing or traditional methods; and to origin-related attributes, such as local or organic production. Despite its conceptual ambiguity, naturalness is commonly associated with healthiness, freshness, stability, and tastiness [129]. Perceptions of unnaturalness are subjective, influenced by culture and religion, and vary across countries [130,131]. Technological framings may increase perceived artificiality and unnaturalness, which in turn can predict disgust [129,132]. These aspects are particularly relevant in Poland, where consumers scored highest in the EU for concerns about food additives [133], suggesting increased susceptibility to frames that present cultured meat as artificial or excessively technological. Accordingly, Poles reporting stronger concerns about technology and naturalness expressed lower acceptance of cultured meat [21].

Taken together, the main barriers to cultured meat acceptance in Poland do not appear to result solely from limited knowledge of cultured meat itself. They also reflect broader beliefs about science, technology, food production, and naturalness. In the long term, broad educational initiatives that improve science literacy and trust in science may support acceptance, though such interventions require sustained structural effort [124,128]. In the shorter term, increasing familiarity with cultured meat remains essential. Communication strategies should avoid unnecessary technology-centered associations because non-technical nomenclature can reduce perceived unnaturalness and disgust. Positive framing may also reduce the novelty effect and increase acceptance [128]. More specifically, framing cultured meat as analogous to animal flesh can decrease disgust [132], whereas consumption-oriented framing can increase expectations of naturalness, taste, and familiarity, thereby improving attitudes [134]. Nevertheless, communication should be balanced. Because cultured meat remains at an early stage of development, overly positive framing may inflate expectations and obscure plausible limitations in sensory, nutritional, regulatory, or price-related areas. This could be counterproductive if consumers attracted by optimistic messaging later encounter product imperfections, especially among sensory-oriented consumers [21,25]. Transparent information about production processes and readily accessible safety and quality data may therefore be more effective for building trust than purely promotional communication [124]. Once cultured meat becomes commercially available, product labeling should provide sufficiently detailed information to meet the expectations of diverse Polish consumers, including those with high technology neophobia [94].

### 4.3. Acceptance of Plant-Based Meat Alternatives

Acceptance of plant-based meat alternatives among Poles has been explored in six studies [12,96,97,98,99,100], one of which focused exclusively on meat excluders, namely vegetarians and vegans [96], and another study involved acceptance comparison among meat eaters and meat reducers [100]. Studies involving omnivores were generally more diverse in terms of gender, age, and education [12,97,99]. Because plant-based meat alternatives are already commercially available, acceptance was often assessed using indicators such as purchase frequency, consumption frequency, and intention to consume more, rather than only hypothetical willingness to try [12,97,99]. In one study, acceptance was examined by comparing various attributes of plant-based meat alternatives to meat. Overall acceptance was low to moderate [12,98,99,100]. While familiarity should not be assumed solely based on market availability, Polish studies indicated that more than one-third of respondents were unaware of these products [12,97]. The most common barriers were high price, unattractive taste, and concerns about low nutritional value [12,96,98,100]. Importantly, the definition of plant-based meat alternatives may vary across studies, e.g., with respect to whether pulses are included as alternatives or whether animal-derived ingredients, such as eggs or milk, are included. Only one analyzed study detailed an adapted definition [99].

Meat eaters and meat excluders or reducers perceive plant-based meat alternatives differently in terms of their properties; importantly, perceptions also vary among meat eaters depending on their broader attitudes toward meat and food [98,99,100]. Meat excluders, meat reducers, meat opponents, and conscious food enthusiasts tended to perceive plant-based alternatives as beneficial for health, in contrast to meat lovers, traditionalists, and careless food lovers [96,98,99,100]. These outcomes may stem from the considerable nutritional variability in plant-based alternatives, which depends on ingredients and can, in some products, result in sodium or saturated fatty acid levels exceeding those found in meat. They may also reflect beliefs that certain ingredients, such as soy, could adversely affect health [135]. Consequently, Polish consumers may be particularly reluctant to accept products perceived as nutritionally inferior, given that trust in alternative proteins, including trust in their quality, reliability, safety, origin, authenticity, label accuracy, honesty, truthfulness, and integrity, was positively associated with behavioral intentions toward plant-based meat alternatives [136].

Price appears to be one of the most consistent structural barriers to wider adoption. Polish consumers frequently reported that plant-based meat alternatives were more expensive than meat [12,96,98], which may limit their use even among consumers willing to reduce meat consumption [12]. Sensory expectations represent another major constraint. Plant-based meat alternatives were often expected to resemble meat in taste and culinary performance [99], yet Polish consumers often did not perceive their sensory quality as comparable to, or as attractive as, meat [12,97,98,99,100]. Plant-based meat alternatives are emerging on the market, and product diversity is increasing; however, prior negative experiences with early-commercialized products may reduce willingness to try other products in this category.

Among Polish meat eaters, perceptions and consumption of plant-based meat substitutes depended strongly on attitudes toward meat. Meat lovers and individuals strongly attached to meat reported the lowest consumption of plant-based meat alternatives. They were also the most skeptical about the sensory and nutritional resemblance of such products to meat [12]. Together with strongly committed traditionalists and careless food lovers who expressed skepticism regarding the environmental burden of meat, this limits the likelihood of adopting plant-based meat substitutes in the near term [99]. Importantly, willingness to consume plant-based meat alternatives should not be equated with willingness to reduce meat consumption. Although 60% of respondents declared willingness to increase their consumption of plant-based meat alternatives, only 42% intended to reduce meat consumption [12]. Similarly, consumption of or willingness to consume plant-based meat alternatives does not necessarily translate into meat-reduction intentions or behavior [12,99]. In a cluster-based study, meat reduction in favor of plant-based meat alternatives occurred primarily among conscious food enthusiasts, who also showed the highest engagement in sustainable and ethical food-consumption behaviors [99].

The Internet served as a primary source of information about plant-based meat alternatives, particularly among younger Poles [98]. Online communication may therefore be useful for presenting the culinary utility of meat-like plant-based products and reinforcing positive perceptions, including through food influencers [98]. However, promotion should not rely only on sustainability claims, since environmental and ethical motives were not significantly associated with behavioral intentions to consume plant-based meat alternatives in Poland [136]. Familiarity with and belief in the harmful environmental impact of animal farming may affect the perceived importance of meat reduction and, by extension, the consumption of plant-based meat alternatives, but these motives are likely to be insufficient for consumers whose choices are primarily driven by price, taste, convenience, or attachment to meat [99]. Considering that meat excluders were particularly interested in products that could serve as bases for main courses, including plant-based substitutes for minced meat, gyros, chicken-like pieces, and burgers [96], communication aimed at meat eaters may be more effective when it presents plant-based meat alternatives as direct substitutes for familiar meat-based dishes. Additionally, increasing exposure to plant-based meat alternatives could encourage moderate or undecided consumers, particularly if such products are offered as regular options in hospitals, school cafeterias, or restaurants. Also, given that Christianity remains the dominant religion in Poland and Friday meat abstinence is a recognizable cultural practice, offering plant-based meat alternatives in cafeterias on that day may provide an additional culturally familiar opportunity for consumers to encounter these products [137].

### 4.4. Acceptance of Edible Insects

Acceptance of edible insects among Poles has been examined more extensively than acceptance of most other alternative protein sources, with twelve studies involving diverse samples. However, the available evidence remains constrained by sample limitations. Young adults often dominated study groups [101,102,103,107,109,110,116], along with women [101,102,103,104,107,109,110,116], urban residents [103,104,110], and university students or individuals with higher education [102,103,104,110,116]. Acceptance was measured mainly as willingness to try, consume, or purchase edible insects, or as a general attitude towards them [101,102,104,106,108,116], as well as comparison to conventional meat [100]. The available studies consistently indicate low acceptance of insects as food among Poles [101,104,108,109], despite relatively high awareness that insects can be used as a food source [101,104,106,107,116]. Factors negatively associated with acceptance include female gender [101,104,108], food neophobia, disgust, low openness to experience, and concerns about sensory properties [100,101,102,104,106,108,110,116]. The most frequently reported positive motives or predictors were curiosity and environmental concerns, including the perceived potential of edible insects to reduce environmental impact [101,103,104,116].

Edible insects are not embedded in Polish culinary traditions, unlike in regions with established histories of entomophagy. They are also classified in the European Union as novel foods. Food novelty creates an informational gap concerning use, sensory characteristics, nutritional value, and safety. Poles reported spending little or no time seeking information about insect-based foods [108], which is consistent with poor to moderate awareness that edible insects may serve as an alternative to meat and dairy products [104], and limited awareness of their environmental advantages [108]. In the absence of specific food-related knowledge, consumers may rely on the most readily available associations with insects in general. In populations without a history of insect consumption, including Poland, insects are often associated with pests, contamination, disease transmission, or health hazards [108,116,138]. This may explain why Poles did not generally perceive edible insects as healthy, safe, or nutritious, and why safety concerns emerged as a barrier to acceptance [104,108]. Also, these associations help explain the central role of disgust in the rejection of edible insects. Disgust is an affective response to an offensive object that produces revulsion [139] and is thought to support avoidance of potential pathogens or toxins [140]. At the societal level, this response can contribute to classifying edible insects as culturally inappropriate foods [116,141]. The strength of these responses varies across individuals. For example, women tend to show greater sensitivity to disgust [142,143], whereas individuals with high food neophobia and low openness may be particularly reluctant to consume unfamiliar foods, such as edible insects. Unfamiliarity with edible insects as food can also reduce expected sensory liking [144,145]. Together, disgust, unfamiliarity, and perceived cultural inappropriateness may explain why many Poles rejected the idea of trying a dish containing insects, even if prepared by famous chefs [108], and why cognitive heuristics and biases remain powerful barriers to acceptance [50,144].

Strategies aimed at increasing acceptance should therefore address both knowledge and hedonic expectations. Greater familiarity with edible insects and their qualities may reduce disgust and increase acceptance [146], but communication should also emphasize sensory and culinary properties [147]. This is important because, among Poles willing to try insect-based products, curiosity appears to be a major motivation [101,104,116], probably reflecting variety-seeking behavior rather than a stable, utilitarian commitment to long-term dietary change [50,106]. Increasing acceptance of edible insects in Poland is therefore likely to require a broader educational and promotional strategy. A single positive tasting experience may not be sufficient if the product category is still perceived as culturally inappropriate [116,144]. Broader knowledge and familiarity with edible insects can support attitude change and influence behavioral outcomes such as willingness to try or purchase specific products [102,104,108,110,148], partly by weakening pest-related frames [50]. Polish consumers should therefore be provided with clear information about the lower environmental impact of insect production compared with meat and the nutritional density of edible insects. Such information can support cognitive evaluation of distant benefits and contribute to framing edible insects as a healthy and sustainable food choice [149]. Communication may be delivered through multiple channels, including social media, product labels, and expert recommendations [150]. These messages may be particularly relevant for younger Poles concerned about the environment and health [102,103]. Safety communication requires particular care. Transparency regarding farming, manufacturing, and scientific evidence may help mitigate safety concerns [101,102,108], and informational campaigns should address this issue [110]. However, making safety concerns the central theme of public communication may unintentionally reinforce the frame that edible insects are primarily a safety hazard [151]. This is especially relevant because pragmatic consumers with low acceptance of insect-based products may simultaneously report great concern about food safety [102]. Alongside utilitarian messages, hedonic messages may be needed to reach more pleasure-oriented consumers by highlighting immediate culinary benefits [145]. Celebrity endorsement may be particularly effective for delivering hedonic messages when social support for insect consumption is low [147,152].

Because food neophobia and low openness to experience are important barriers in Poland, early communication strategies should be targeted primarily at potential early adopters [101,102,104,110]. In Poland, these may include men [101,108], younger individuals with weaker attachment to traditional Polish cuisine, consumers receptive to improved nutritional density and manufacturer certification [110], individuals with higher material status and education [108], and those with low food neophobia [101]. Such groups may form an initial consumer niche that gradually reduces the perceived social inappropriateness of eating insects. This is important because social exposure, including observing others consuming insects, can reduce disgust and increase acceptability [153].

Product-specific factors are equally important. Among the minority of Poles who had previously eaten insects [101,104,106,109], taste was often rated as good or neutral [104,109,114]. This suggests that rejection may be driven more by anticipated disgust than by actual sensory experience. Products should therefore minimize psychological triggers and align with Polish sensory preferences. Poles preferred insect flour or ground insects over whole insects, as the absence of visible insect morphology reduces cues that trigger aversion [53,101,109,116]. Incorporating insect flour into familiar Polish dishes, especially dough-based products, may increase acceptance by embedding the ingredient in a culturally recognizable food matrix [116,144,154]. Insect flour is protein-rich, gluten-free, and introduces distinctive flavor notes; depending on the level of inclusion, it may also alter the sensory and technological properties of pasta, bread, and other dough-based products [50,53]. Because sensory attributes remain decisive for Polish acceptance of insect-based foods [101], particularly among pleasure-oriented consumers, manufacturers should conduct pre-market sensory and focus-group testing to optimize recipes [144]. This is necessary because sensory penalties may depend on both the insect species and the fortified product matrix [50]. Product-centered Polish studies indicate that bread containing 10% house cricket flour may be acceptable [53], as well as muffins with 4% house cricket flour [118], bars with 7.4% ground mealworms [105], wheat pancakes with 10% house cricket/mealworm/buffalo worm flour [114], or wheat sponge cake with 10% mealworm flour, but further Polish sensory studies are advocated before broader product-specific conclusions are drawn. Finally, for some younger Poles, convenience and ease of use are becoming increasingly important, suggesting that ready-to-prepare products may be a more promising format than ingredients requiring substantial culinary effort.

### 4.5. Acceptance of Microalgae

Information on consumer acceptance of microalgae among the Polish population is very limited and should be interpreted cautiously. Several Polish product-development studies included sensory evaluation of products containing microalgae [118,119,120,121]. However, these studies primarily assessed specific product formulations, composition, or technological properties rather than consumer attitudes towards microalgae as a food category. They also differed in product type, microalgae content, and sensory-assessment design. Therefore, they should be treated as indirect evidence only, not as full consumer-acceptance studies. Nevertheless, these studies indicate that microalgae-containing foods may achieve acceptable sensory scores among Polish consumers when incorporated at relatively low levels and in familiar food matrices. For example, shortbread biscuits supplemented with 1–6% Spirulina powder were still acceptable to consumers despite the darker color and increased hardness compared with controls [120]. Similarly, vegan muffins containing 0.5–1.5% microalgae were accepted in terms of appearance, taste, and overall quality [121]. In another study, spirulina-enriched vegan basil pesto was rated favorably, particularly at 1% addition, whereas increasing the level to 2% reduced the overall score but did not make the product unacceptable [119]. A study comparing muffins enriched with house cricket or spirulina powder also suggested that spirulina could improve nutritional value while maintaining acceptable taste, with spirulina muffins receiving particularly high texture scores [118].

The only Polish study directly addressing consumer experiences with microalgae was a small-scale online survey of current users of microalgal food supplements, including Spirulina, Chlorella, and Aphanizomenon products (n = 150) [72]. As the sample consisted exclusively of previous or current supplement users, the study cannot be used to infer acceptance among the general Polish population, non-users, or prospective consumers. Nevertheless, it provides useful insight into the consumer niche already engaged with microalgae. Participants used these products mainly for nutritional purposes, immune support, and detoxification, suggesting that microalgae were perceived primarily through a health-promoting or functional-food lens rather than as conventional food ingredients. The Internet was the dominant source of information, while consultation with health professionals was uncommon, indicating that consumer knowledge may be strongly shaped by online claims and marketing. Importantly, the study also assessed satisfaction and willingness to recommend products, which are relevant proxies for post-use acceptance. Most Spirulina and Chlorella users reported satisfaction and would recommend these supplements, whereas Aphanizomenon users reported very low satisfaction and no perceived beneficial effects. At the same time, the relatively frequent self-reported adverse events, particularly among Aphanizomenon users, indicate that safety experience may be an important determinant of continued acceptance [72]. Overall, this study suggests that Polish acceptance of microalgae may currently be concentrated among health-oriented consumers and supplement users, but broader acceptance as a food category remains insufficiently studied. In summary, no Polish study has comprehensively examined microalgae acceptance among the general population or among prospective users of microalgae-based foods. Sensory ratings in product-oriented tasting studies tended to decrease as microalgae content increased [120], but these findings should not be overinterpreted as evidence of general consumer acceptance or rejection. Future studies should distinguish between acceptance of microalgae as supplements and as food ingredients, assess familiarity and perceived health or safety concerns, and include representative or at least demographically diverse samples.

### 4.6. Acceptance of Mycoproteins

Only one small-scale, online, non-representative study (n = 240) has examined mycoprotein acceptance among Poles, specifically in relation to Quorn™ products [122]. Consequently, general acceptance of mycoprotein in Poland cannot be reliably assessed. The responses nevertheless suggest limited acceptance: most participants either answered negatively or were unsure whether they would buy products labeled as “microbiological”. The highest acceptance was observed among meat excluders (n = 66). These results should be interpreted with caution. The term “microbiological” may itself have shaped responses and may not capture acceptance of mycoprotein when presented as a meatless, vegetarian, fungal-protein, or high-protein food. The findings may therefore reflect poor familiarity with mycoprotein’s food-related properties and its production process, or the effect of unfamiliar or unattractive terminology. The authors argued that products such as Quorn™ should be labeled as “meatless” or “vegetarian” rather than “microbiological” to increase on-shelf acceptance [122]. Overall, the available evidence is therefore too limited to determine whether low acceptance reflects rejection of mycoprotein itself, low consumer familiarity, or the specific terminology used to describe these products.

## 5. Recommendations and Future Research Directions

**Figure 1:**
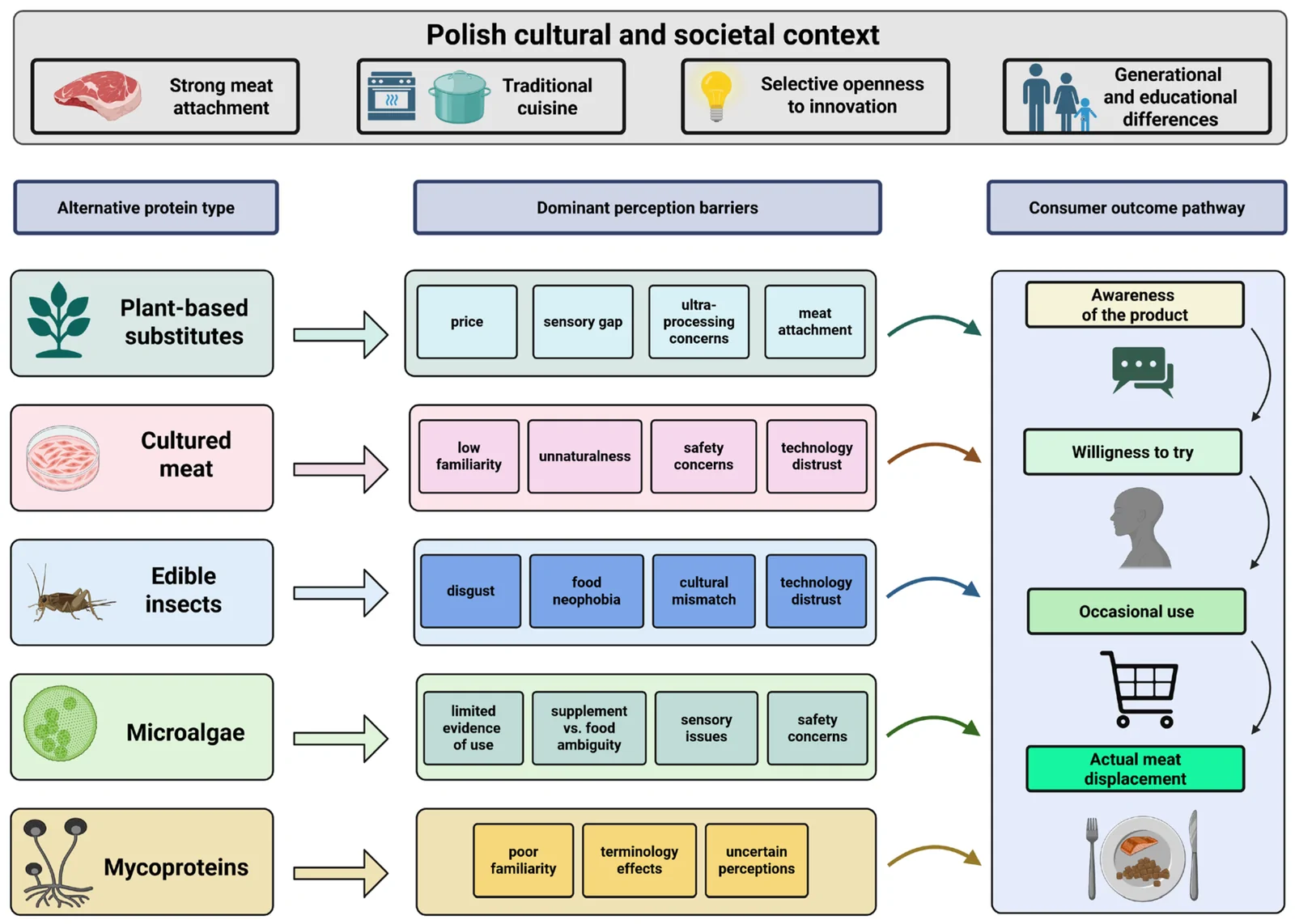
Conceptual framework of category-specific determinants of alternative protein acceptance in Poland. The figure illustrates that consumer acceptance of alternative proteins is shaped by both product-specific barriers and the broader Polish food culture. Rather than representing acceptance as a uniform response to a single category of “alternative proteins”, the framework distinguishes between protein sources and shows how different perception filters may influence progression from awareness to trial, repeated use, and potential meat displacement. The model emphasizes the need for category-specific research, communication, and product-development strategies. Graph created with BioRender.com.

The current evidence indicates several directions for future research and for the development of communication strategies concerning alternative protein sources in Poland. First, future studies should use larger and more representative samples that better reflect the demographic structure of the Polish population. This is particularly important because many available studies relied on self-selected online samples, often overrepresenting women, younger adults, urban residents, students, and individuals with higher education. Such sampling patterns may lead to overestimation of awareness, sustainability-oriented attitudes, or openness to food innovation. Second, research on alternative proteins in Poland would benefit from greater methodological standardization. Existing studies used different self-designed questionnaires and heterogeneous measures of acceptance, including willingness to try, consume, purchase, or recommend a product, as well as broader attitudinal indicators. This limits direct comparison across product categories and over time. Future studies should consider using validated tools to assess food neophobia, disgust sensitivity, naturalness perception, technology neophobia, environmental concern, meat attachment, trust in science, and perceived safety. Third, the evidence base remains dominated by quantitative survey designs. Qualitative and mixed-methods research is needed to better understand how Polish consumers interpret alternative proteins; what meanings they attach to “naturalness”, “processing”, “safety”, “sustainability”, and “meat replacement”; and how these meanings differ between consumer segments. Such approaches may be particularly useful for studying novel or poorly familiar categories, including cultured meat, edible insects, microalgae, and mycoprotein. Fourth, more product-specific and context-specific studies are needed. Acceptance of alternative proteins is unlikely to depend only on the source of protein itself, but also on product format, sensory properties, culinary familiarity, price, labeling, availability, and perceived fit with everyday meals. This is especially relevant for edible insects and microalgae, for which incorporation into familiar food matrices may reduce psychological barriers and improve sensory acceptance. Similarly, plant-based meat alternatives should be studied not only as a broad category but also according to product type, nutritional profile, degree of processing, and intended culinary use. This product-specific perspective is synthesized conceptually in Figure 1, which links category-dependent perception filters with the broader Polish food-culture context and successive stages of consumer acceptance. Fifth, future research should distinguish between declared acceptance and actual behavior. Willingness to try or purchase alternative proteins does not necessarily translate into repeated consumption or meat reduction. Longitudinal studies, choice experiments, sensory trials, and real-market studies would therefore be valuable for assessing whether initial curiosity or positive attitudes lead to sustained dietary change.

## 6. Conclusions

Research on consumer acceptance of alternative protein sources in Poland remains fragmented and unevenly distributed across product categories. Edible insects and cultured meat have received the greatest attention, whereas evidence on microalgae and mycoprotein remains particularly limited. Plant-based meat alternatives are more familiar and well established, but their acceptance is often constrained by price, sensory expectations, perceived nutritional quality, and concerns about processing. Across alternative protein categories, Polish consumers appear to respond not only to sustainability or ethical arguments, but also to more immediate and personally relevant factors, including taste, safety, naturalness, price, convenience, and familiarity. The evidence suggests that environmental benefits alone are unlikely to be sufficient to drive broad acceptance, especially when products are perceived as unfamiliar, artificial, highly processed, culturally inappropriate, or sensorially inferior to conventional foods. The reviewed studies also show that acceptance is not uniform across the Polish population. Younger consumers, individuals with higher education, and those with lower food neophobia, greater environmental concern, lower attachment to meat, and greater familiarity with alternative proteins tend to be more receptive. However, the predominance of non-representative and survey-based studies limits the generalizability of these findings. Alternative proteins may contribute to dietary diversification and more sustainable food systems in Poland, but their wider acceptance will depend on product-specific improvements, transparent communication, culturally appropriate framing, and a stronger evidence base derived from representative, comparative, and methodologically robust studies.

## Figures and Tables

**Table 1:** Summary of Polish studies on consumer acceptance of alternative protein sources. Findings were harmonized into brief statements to improve comparability across heterogeneous study designs and outcomes.

Type	Year	Sample Size	Age (Years)	Female/ Male [%]	Main Finding	Reference
**Cultured meat**	2023	1553	38.3 ± 13.4 (mean ± SD)	78/21	Moderate openness: 54% willing to purchase; 63% familiar with cultured meat.	[25]
**Cultured meat**	2021	424	Range: 19–25 (54%) 26–30 (17%) 12–18 (13%) 31–40 (10%) 41–50 (2%) 51–60 (2%) 60+ (2%)	75/24	Moderate openness: 55% of women and 63% of men willing to try.	[93]
**Cultured meat**	2026	425	Range: 18–34 (42%) 35–54 (41%) 54+ (17%)	66/34	Moderate acceptance; the largest groups were cautious optimists and concerned ambivalents.	[21]
**Cultured meat**	2023	1286	40.4 ± 12 (mean ± SD) Range: 18–89	52/47	Label priorities centered on expiry date and ingredients; the in vitro marking was less important.	[94]
**Cultured meat**	2023	418	Range: Under 18 (11%) 18–26 (57%) 27–35 (20%) 36–45 (6%) 46–55 (6%)	72/22	High willingness to try: 34% gave the maximum score (7/7), and another 34% gave 5–6/7.	[95]
**Plant-based**	2025	220	Range: 18–25 (28%) 26–35 (36%) 36–45 (22%) 46–55 (9%) 56–65 (4.5%) 66+ (0.5%)	85/13	Among vegans/vegetarians, 63% viewed plant-based substitutes as healthier; 70% were satisfied with availability.	[96]
**Plant-based**	2025	1003	45.4 ±15.6 (mean ± SD) Range: 18–24 (10%) 25–34 (19%) 35–44 (20%) 45–54 (16%) 55–64 (22%) 65+ (12%)	52/48	60% were willing to increase plant-based product intake, but 58% did not plan to reduce meat consumption.	[12]
**Plant-based**	2023	1003	Range: 18–24 (10%) 25–34 (20%) 35–44 (20%) 45–54 (16%) 55–64 (22%) 64+ (12%) Range: 18–83	52/48	Attitudes were broadly non-negative; 47% consumed plant-based meat and most users did not find it tasteless.	[97]
**Plant-based**	2026	263	Range: 18–25 (81%) 26–35 (11%) 36–45 (6%) 46–56 (2%)	74/26	Among mostly young respondents, 58% of non-vegans had bought a vegan product; 37% were interested in meat alternatives.	[98]
**Plant-based**	2025	1200	Range: Under 24 (12%) 25–34 (18%) 35–49 (24%) 50+ (46%)	54/46	Use was occasional: 14% bought plant-based meat weekly or more; 24% never bought it.	[99]
**Plant-based,** **edible insects, cultured meat**	2025	1016	Adults	51/49	Plant-based alternatives were the most favorable among tested meat alternatives. The perception varied depending on meat-related dietary habits, where meat eaters assessed all substitutes less positively than meat reducers.	[100]
**Edible** **insects**	2020	464 survey	18–24	65/35	Low acceptance: 59% would not accept insects in their diet.	[101]
**Edible** **insects**	2020	402 testing	18–78	No data	Tasting increased openness, suggesting that positive sensory experience may improve acceptance.	[101]
**Edible** **insects**	2025	947	Students	60/40	Highest acceptance was reported by 33%; sustainability, safety, nutrition and sensory appeal shaped acceptance.	[102]
**Edible** **insects**	2024	950	Generation Z	60/40	Generation Z showed greater acceptance when insects were incorporated into familiar ready-made products.	[103]
**Edible** **insects**	2025	1553	38.3 ± 13.4 (mean ± SD)	78/21	Awareness was high (98%), but purchase willingness was low (28%).	[104]
**Edible** **insects**	2020	101	Students	73/27	Visible insects and texture were stronger barriers than taste; hidden mealworm flour was more accepted.	[105]
**Edible** **insects**	2021	1096	44 ± 15.15 (mean ± SD) Range: 16–78	55/45	Awareness was common, but experience was rare; acceptance was higher among men and environmentally aware consumers.	[106]
**Edible** **insects**	2025	70	Range: 18–25 (62%) 26–35 (23%) 36–45 (3%) 46–55 (7%) 56–65 (1%) 65+ (3%)	70/30	Prior consumption was similar in Poland and Spain; Polish participants showed greater willingness to consume insect-enriched foods.	[107]
**Edible** **insects**	2025	631	Range: Under 29 (18.5%) 30–49 (40%) 50+ (42,5%)	52/48	Knowledge and perceived benefits were limited; aversion remained moderate to high.	[108]
**Edible** **insects**	2024	790	Range: 18–26 (70%) 27–35 (17%) 36+ (13%)	89/8	Acceptance varied by diet: lowest among vegans, highest among pescatarians and flexitarians.	[109]
**Edible** **insects**	2024	1063	Students	61/39	Lower food neophobia and perceived health-promoting quality increased purchase intention.	[110]
**Edible** **insects**	2024	106	Students Range: 20–27	No data	Insects were mostly associated with protein and sustainability, but perceived safety was limited.	[111]
**Edible** **insects**	2022	50	Range: 19–51	No data	Sponge cake with 10–20% mealworm flour was less accepted than the control but still rated moderately.	[112]
**Edible** **insects**	2024	749	Students Generation Z	55/45	Product type and consumer profile mattered: men preferred ready meals/baked goods; health-aware women preferred supplements/drinks.	[113]
**Edible** **insects**	2023	60	Range: 19–23	No data	The amount of addition insect flour in pancakes had a statistically significant effect on sensory evaluation, but the insect species had no such effect.	[114]
**Edible** **insects**	2023	141	Young adults (18–29) and seniors (>65)	No data	The most preferable insect was mealworm as a 20% additive to cream-type veggie soup. No statisitcal differences were observed between age groups.	[115]
**Edible** **insects**	2020	99	Range: 18–45	82/18	Explicit insect labelling increased reluctance and decreased consumed quantity, regardless of food type.	[116]
**Edible** **insects**	2024	41	Students	66/34	The control pastry was rated highest; insect addition lowered sensory scores, especially color and aroma.	[117]
**Edible** **insects**	2025	70	Range: 19–36	67/33	Cricket muffins had lower color, aroma, and overall scores; taste did not differ significantly from other muffins.	[118]
**Microalgae**	2025	70	Range: 19–36	67/33	Spirulina muffins scored highest for texture; taste did not differ significantly across muffin variants.	[118]
**Microalgae**	2017	150	34.6 ± 10.8 (mean ± SD)	76/24	Satisfaction differed by supplement: Spirulina and Chlorella were moderately accepted, and Aphanizomenon was poorly accepted.	[72]
**Microalgae**	2024	35	Range: 21–45	66/34	Low-level spirulina in pesto was acceptable; 1% scored highest, while 2% reduced acceptance due to color.	[119]
**Microalgae**	2017	30	Range: 23–53	No data	Biscuits with 0–1% spirulina were most acceptable; higher addition and storage reduced acceptance.	[120]
**Microalgae**	2023	20	Range: 18–25 (95%) 26–30 (5%)	85/15	Chlorella addition did not significantly affect sensory scores but caused a grassy aroma and a darker color.	[121]
**Mycoproteins**	2017	240	Range: Under 18 (3%) 18–29 (76%) 30–50 (19% 50+ (2%)	74/26	Acceptance of mycoprotein was higher among meat excluders than meat eaters; many participants were undecided.	[122]

SD means standard deviation.

**Table 2:** The most prominent factors and barriers to the acceptance of different types of alternative protein sources.

Alternative Protein	Promoting Factors	Barriers
**Cultured meat**	Lower environmental impact and improved animal welfare	Safety concerns, perceived unnaturalness, and limited knowledge
**Plant-based**	Market availability	Lack of awareness of these products, high price, unattractive taste, concerns about low nutritional value
**Edible insects**	Curiosity, lower environmental impact, incorporation at relatively low levels and in familiar food matrices	Female gender, food neophobia, disgust, low openness to experience, concerns about sensory properties, higher content in food matrices
**Microalgae**	Incorporation at relatively low levels and in familiar food matrices	Higher content in food matrices
**Mycoproteins**	Reduced meat consumption	Poor familiarity with mycoprotein food

SD—standard deviation.

## Data Availability

Data sharing is not applicable. No new data were created or analyzed in this study.
